# Modelling antimicrobial resistance transmission to guide personalized antimicrobial stewardship interventions and infection control policies in healthcare setting: a pilot study

**DOI:** 10.1038/s41598-023-42511-5

**Published:** 2023-09-22

**Authors:** Francesco Durazzi, Maria Diletta Pezzani, Fabiana Arieti, Omar Simonetti, Lorenzo Maria Canziani, Elena Carrara, Lorenzo Barbato, Francesco Onorati, Daniel Remondini, Evelina Tacconelli

**Affiliations:** 1 Division of Infectious Diseases, Department of Diagnostics and Public Health, University of Verona, Verona, Italy; 2 Department of Physics and Astronomy, University of Bologna, Bologna, Italy; 3 Infectious Diseases Unit, University Hospital, Trieste, Italy; 4 Department of Pharmacy, Azienda Ospedaliera Universitaria Integrata Verona, Verona, Italy; 5 Department of Cardiac Surgery, Verona University Hospital, Verona, Italy

**Keywords:** Infectious diseases, Health policy, Computational science

## Abstract

Infection control programs and antimicrobial stewardship have been proven effective in reducing the burden of diseases due to multidrug-resistant organisms, but quantifying the effect of each intervention is an open issue. For this aim, we propose a model to characterize the effect of interventions at single ward level. We adapted the Ross-Macdonald model to describe hospital cross-transmission dynamics of carbapenem resistant *Klebsiella pneumoniae* (CRKP), considering healthcare workers as the vectors transmitting susceptible and resistant pathogens among admitted patients. The model parameters were estimated from a literature review, further adjusted to reproduce observed clinical outcomes, and validated using real life data from a 2-year study in a university hospital. The model has been further explored through extensive sensitivity analysis, in order to assess the relevance of single interventions as well as their synergistic effects. Our model has been shown to be an effective tool to describe and predict the impact of interventions in reducing the prevalence of CRKP colonisation and infection, and can be extended to other specific hospital and pathological scenarios to produce tailored estimates of the most effective strategies.

## Introduction

Antimicrobial resistance (AMR) poses a worldwide public health concern that undermines the provision of effective treatments, leading to limited and more harmful therapeutic options and increased risk of death^1^. In 2019, 1,27 million deaths have been attributed to AMR globally^1^. Notably, over 80% of newly approved antibiotic agents are developed from current classes where resistance mechanisms are well-established and rapid emergence of resistance is foreseen, thus limiting their clinical benefit^2^. The constant increase of AMR results from an interplay of several drivers, ranging from human and animal antimicrobial misuse or overuse, healthcare transmission, suboptimal availability of diagnostics and vaccines^3^. The availability of surveillance data of AMR is a key component of antimicrobial stewardship (AMS) and infection prevention and control (IPC) policies, which have proved to be successful in decreasing resistance rates and improving patients’ outcomes^4–8^. The availability of large datasets and the development and introduction of new complex algorithms and computer implementable instructions (i.e. artificial intelligence and machine learning), can contribute to enhance surveillance activities and consequently implement AMS programs by identifying targets for improvement and tailoring specific interventions^9^.

Mathematical models that estimate cross-transmission of multidrug-resistant (MDR) bacteria in the healthcare setting are important tools that further enforce AMR surveillance. The added value of these models is the potential to predict the effectiveness of IPC and AMS programs, as a single intervention or as a bundle in reducing the rates of MDR pathogens^10,11^. Firstly conceptualized in the early decades of the XX century to study vector born disease and contagious epidemics^12,13^, evolutionary epidemiology models have progressively flourished from the 90s in the attempt to investigate the epidemiological changes in the bacterial populations, as well as the evolution and transmission of resistant strains^14,15^. In the last decades, cross-transmission models for MDR pathogens have been increasingly employed in the context of healthcare-associated infections (HAI), although with conflicting results^11,16,17^. Crucial issues to fit evolutionary model outcomes with reality are modelling the competition between resistant and susceptible strains and their coexistence (as they are not always mutually exclusive), co-colonization with other bacterial strains/species, presence of not colonised patients, active multimodal policies and implementation of new IPC and/or AMS strategies. Consequently, shortcomings include availability of clear parameters such as information on asymptomatic carriage, timing of events (e.g. infection), transmission rates between compartments, heterogeneity in the host populations, strain typing and quantified impact of hand hygiene frequency, antibiotic consumption, and screening strategies^18–20^.

The primary objective of this study was to design and validate a mathematical transmission model able to predict the potential impact of several AMS and IPC strategies in reducing the prevalence and/or infection of target MDR bacteria within hospital setting. We considered a case study for carbapenem resistant *Klebsiella Pneumoniae* (CRKP) but the modelling framework can be extended to other pathogens. Few modelling studies have focused on CRKP transmission^17,21,22^. In these studies, drug-susceptible strains of KP are neglected, and only isolation, hand hygiene compliance and contact precautions are modelled to assess the impact of infection control measures. A recent study^22^ examined the impact of newly admitted colonized patients on the endemic prevalence of CRKP and evaluated the effect of antibiotic treatment on transmission, but no clinical data were used to estimate the associated parameters or to validate the model.

The second objective was to assess and compare the effect size of different pairs of AMS and IPC interventions. To address the major drawbacks reported above, we (i) considered three different compartments for patients without the target bacteria and with the susceptible or resistant strains, respectively, (ii) proposed and validated model parameterization from real clinical data, (iii) assessed the ability of the model to describe longitudinal point prevalence data, and iv) quantified the impact of different interventions, singularly and in combination, through sensitivity analysis.

## Material and methods

The model was developed through a multistep approach. First, we carried out a scoping review of studies quantifying the effect of IPC and AMS interventions on the prevalence and/or incidence of colonisation and/or infection due to the critical resistant bacteria of the WHO Pathogen Priority List (PPL) for research and discovery of new effective antibiotics^23^. Second, a modified Ross-Macdonald model^12^ was developed. The model was validated using epidemiological data prospectively collected during an intervention of AMS and IPC procedures (before and after study) implemented in a 1500-bed Italian teaching hospital^24^. Finally, we run through the model multiple scenarios of single or combined interventions to estimate their different impact on the prevalence of the target MDR bacterium.

### Scoping review

To identify the most relevant interventions to be included in the model as described in literature, we considered systematic reviews published in English language from 01.01.2010 to 31.03.2021, focusing on AMS and IPC interventions to reduce incidence and/or prevalence of target pathogens. Search terms and forms for data collection are reported in Supplementary Tables 1 and 2. We searched information for methicillin-resistant *Staphylococcus aureus* (MRSA), Vancomycin-resistant *Enterococci* (VRE), Carbapenem-resistant *Enterobacterales* (CRE), including CRKP, carbapenem-resistant *Pseudomonas aeruginosa* (CRPA) and Carbapenem- *resistant Acinetobacter* *baumannii* (CRAB). Systematic reviews analysing the following single interventions, or a bundle including at least two of them, were considered: antibiotic cycling, audit and feedback, staff or patients cohorting, isolation (including pre-emptive), decolonisation, hand hygiene, environmental cleaning, active surveillance (e.g. universal screening, targeted screening or targeted and weekly screening).

Comments, reports, position papers, articles based on questionnaires or ethical implications, pure cost-effectiveness analysis of interventions, national surveys, description of outbreaks, bacterial airborne or respiratory shedding as well as contamination during surgery procedures, environmental and/or clothes sampling prevalence, quality of care and satisfaction among isolated patients were excluded.

### Transmission model

**Figure 1:**
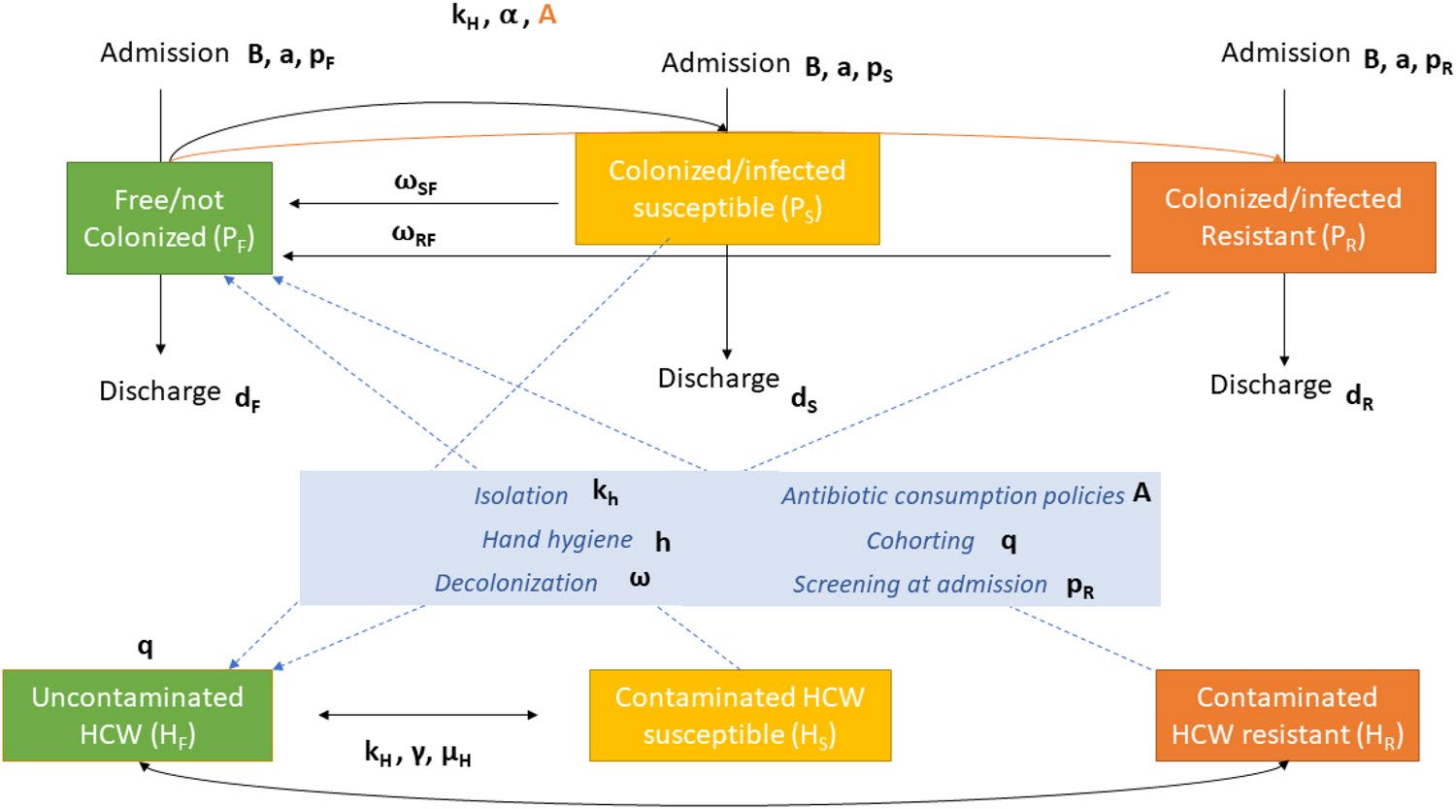
Transmission model: the epidemiological state of a patient belongs to one of three different categories: free/not colonized from the target pathogen (*P*_*F*_), colonized/infected by susceptible strain (*P*_*S*_), colonized/infected by resistant strains (*P*_*R*_). Similarly, healthcare workers (HCWs) can be uncontaminated or contaminated with susceptible or resistant strains (HS and HR respectively).

We based our transmission model on the Ross-Macdonald model for vector-borne diseases^12^. The mathematical model is a system of six differential equations, representing the dynamics of two populations, healthcare workers (HCWs) and patients (P), divided into three compartments based on their epidemiological state [un-colonized or free (F), colonized/infected by susceptible strains (S), and colonized/infected by resistant strains (R)]. We aggregated colonized and infected patients based on sample collection to increase the sample size. Furthermore, since the model focuses on the contamination/transmission dynamics, distinction between colonization and infection has a minor impact, although the model could be extended accordingly to data availability. Figure 1 describes the HCW-patient transmission model and the possible effects of the target AMS and IPC interventions on the transmission dynamics.

The variable P represents the patient population, distinguished in uncolonized (P_F_) and colonized/infected by susceptible (P_S_) and resistant (P_R_) strains, while the variable H refers to the HCW population stratified analogously (H_F,_ H_S_ or H_R_). The cohorting intervention aims at reducing the HCW-patient mixing (e.g. through patient isolation or one-to-one nursing)^16^. In our model, in line with^16^, if the total number of HCWs is H, cohorting is equivalent to an effective number of HCWs equal to $H(1-q)$, where q is the fraction of HCWs assigned to cohorting. Since the model is linear with respect to the HCW variables, we simply rescale the initial condition accordingly: ${H}_{F}+{H}_{S}+{H}_{R}=H(1-q)$.

The equations can be written as:

$$\begin{aligned} & {\delta P}_{F}=-{K}_{H}\alpha A{H}_{R}{P}_{F}-{K}_{H}\alpha {H}_{S}{P}_{F}-{d}_{mean}{P}_{F}+a{P}_{F}+{\omega }_{RF}{P}_{R}+{\omega }_{SF}{P}_{S} \\ & \delta {P}_{S}={K}_{H}\alpha {H}_{S}{P}_{F}-{d}_{mean}{P}_{S}+a{P}_{S}{ - \omega }_{SF}{P}_{S}\\ & \delta {P}_{R}={K}_{H}\alpha {AH}_{R}{P}_{F}-{d}_{mean}{P}_{R}+a{P}_{R}{-\omega }_{RF}{P}_{R}\\ & \delta {H}_{F}=-{K}_{H}\gamma \left(1-h\right){H}_{F}({P}_{S}+{P}_{R})+{K}_{H}h\left({H}_{S}+{H}_{R}\right)\left({P}_{F}+{P}_{S}+{P}_{R}\right)+{\mu }_{H}\left({H}_{S}+{H}_{R}\right)\\ & \delta {H}_{S}={K}_{H}\gamma \left(1-h\right){H}_{F}{P}_{S}-{K}_{H}h{H}_{S}\left({P}_{F}+{P}_{S}+{P}_{R}\right){-\mu }_{H}{H}_{S}\\ & \delta {H}_{R}={K}_{H}\gamma \left(1-h\right){H}_{F}{P}_{R}-{K}_{H}h{H}_{R}\left({P}_{F}+{P}_{S}+{P}_{R}\right){-\mu }_{H}{H}_{R} \end{aligned}$$

As detailed in Table 1, some model parameters were retrieved from the literature or from the clinical data, and might differ in the pre- and post-intervention period (e.g. *A*); *h* and *q* parameters were fitted on longitudinal prevalence data. Values for clearance rates ω_SF_ and ω_RF_,were the only based on assumptions (which in future use cases can be replaced by clinical or literature data ).

**Table 1:** Description of the model parameters and the source of their values.

Symbol	Description	Source	Pre-intervention value	Post-intervention value
H	Number of HCWs during a time-shift	Clinical data	17	17
K_H_	Per-capita contact rate (daily contacts per HCW per patient)	Calculated as a function of *h*^17,37^ (see Supplementary Table S3)	0.776 ± 0.008	0.964 ± 0.009
h	Hand hygiene compliance	Fitted on point prevalence time series	0.855 ± 0.008	0.868 ± 0.008
$A\left(\varepsilon ,\xi \right)$	Increase in transmission probability by antibiotic consumption	Clinical data Literature^16,29^	1.50	1.28
ε	Treatment duration (LOS fraction)	Clinical data	0.23	0.15
ξ	Increased risk of acquiring resistance due to antibiotic pressure	Literature^29^	3.15	2.94
d	Average discharge rate (inverse of LOS)	Clinical data	1/10.6 days^−1^	1/9.2 days^−1^
a	Admission rate (new patients per day)	Clinical data	3.4 per day	3.6 per day
p_F_	PF fraction at admission	Clinical data	0.969	–
p_S_	PS fraction at admission	Clinical data	0.025	–
p_R_	PR fraction at admission	Clinical data	0.006	–
μ_H_	Clearance rate for HCWs (inverse of contamination)	Assuming contamination to last 1 h	24 days^−1^	–
γ	Probability of KP contamination P → HCW per single contact	Literature^17^	0.21	–
α	Probability of KP transmission HCW→ P per single contact	Literature^21^	0.45	–
q	Fraction of HCWs allocated to cohorting	Fitted on point prevalence time series	0.10 ± 0.07	0.10 ± 0.07
ω_RF_	Decolonization rate of PR (inverse of colonization duration)	Literature^18^	1 month^−1^	–
ω_SF_	Decolonization rate of PS (inverse of colonization duration)	Literature^18^	1 month^−1^	–

Parameters value is reported both for the pre- and post-intervention periods.

*HCW* health care workers, *P* patients, *KP* *Klebsiella pneumoniae*, *LOS* length of hospital stay, *P*_*F*_ not colonized/free, *P*_*R*_ colonized/infected by resistant strain, *P*_*S*_ colonized/infected by susceptible strain.

The set of differential equations describe the interactions between compartments by means of three main mechanisms:

(a) HCW-patient contacts: patients interact only with HCWs with a per capita contact rate (K_H_), which describes the number of contacts that a single HCW makes with one patient within one day. A HCW can consequently become contaminated with a certain probability (γ parameter) depending on whether the patient was in the (P_S_) or (P_R_) state, thus becoming (H_S_) or (H_R_), respectively. On the other hand, if a HCW is contaminated, a (H_S_) → (P_S_) or (H_R_) → (P_R_) transmission can happen with a probability described by α. In addition, HCWs contamination is modulated by the term (1 − *h*) indicating the fraction of unprotected contacts (with h being the parameter associated to hand hygiene compliance). A protected contact not only prevents transmission of the pathogen, but also decontaminates the HCW, returning it to the H_F_ compartment. The selection and transmission of a resistant strain can be further influenced by the ward antibiotic pressure described by parameter A, which ultimately depends on the antibiotic consumption^16^. We reduced the contact rate (K_H_) for all the patients to implement the effect of patient isolation.

(b) Admission and discharge: patients enter the ward with a daily rate *a*. When admitted, a patient can be either colonized/infected by resistant strain with probability p_R_, colonized/infected by susceptible strains (p_S_) or uncolonized (p_F_ = 1 − p_S_ − p_R_). The average length of stay (LOS) is *1/d* and it can be calculated from the data as the average over the three compartments weighted on the average fraction of users in each compartment. Estimates of prevalence at admission (Supplementary Table S5) were set to the same values for both pre- and post-intervention period, assuming that the level of resistance in the community did not change significatively over the 2 years of the study.

(c) Clearance of carriage state: patients infected by both susceptible and resistant strains return to the uncolonized state with a rate respectively of (ω_SF_) and (ω_RF_). Similarly, HCWs can move to uncolonized state with a rate defined as (μ_H_). Since no clinical data on clearance of carriage state were available, we set the decolonization rates as those assumed by Blanquart^18^, that considers a slow-paced decolonization dynamics. Noteworthy, increasing the decolonization rate could be used to simulate an increase in the frequency of effective clearance interventions.

(d) Infection control interventions: we considered the effect of cohorting, isolation and pre-emptive isolation, antibiotic consumption, hand hygiene, screening at admission (Table 2). Further information on how the interventions were parametrized is provided in Supplementary Table 3.

**Table 2:** Interventions implemented in the model and their relation with model parameters.

Intervention	Modellization	Effect description
Cohorting^16^	H → (1 − q)H	Decreased effective number of HCWs contributing to pathogen transmission
Isolation and pre-emptive isolation^17^	K_H_	Lower contact rate
Antibiotic consumption policies^16,29^	A = 1 + ε(ξ − 1)	More DOTs increase the risk of colonization by resistant strains
Hand hygiene^17^	*h*	Hand-washing after a contact prevents HCW contamination
Screening at admission	p_R_	More accurate screening reduce the resistance prevalence at admission

### Model validation

For model validation, we used the results of an AMS programme (“Stewardship Antibiotica VErona”- SAVE)^24^. The AMS intervention was carried out in the cardiac surgery ward and consisted of a first monitoring phase, followed by the development of specific antibiotic therapy guidelines, and a second phase characterized by regular consultation and periodic audit and feedback reports. Data were retrieved during the pre-intervention period, from 26/02/2018 to 31/03/2019 March 2019 (weeks 1–57), and in the post-intervention period, from 01/04/2019 to 03/05/2020 (weeks 58–114). SAVE data consisted of longitudinal prevalence time series at weekly resolution (details on the clinical setting and the intervention are in Supplementary Tables 4 and 5, and Supplementary Fig. 1). The MDR bacteria chosen for model validation was CRKP. Cultures from surveillance and clinical activities were collected within 72 h upon admission and recorded on a weekly basis. More details on sample collection and definitions in Supplementary. Samples yielding negative results for both CRKP and CSKP were defined as “free”.

For model validation, we aimed at reproducing the longitudinal prevalence data in both pre- and post-intervention periods. The SAVE data in the pre- and post- intervention periods provided both the input (parameters) and the output (prevalence data) to the model. Specifically, we used the model to reproduce the effect of reduced consumption of different antibiotic classes for which the days of treatment (DOT) pre- and post-intervention have been calculated (Supplementary Methods). Parameters not available from literature or SAVE data were fitted on the longitudinal data using the Levenberg–Marquardt algorithm and refined with likelihood maximization via Monte-Carlo Markov Chain through *emcee* (Python package lmfit)^25^. The parameters of the model are defined on a daily scale (e.g. number of admissions per day), while the point prevalence data were provided on a weekly scale. In order to compare model and data, the model output was sampled on a weekly scale.

Based on the values of the model parameters, multiple model outputs can be produced, some of which represent the interventions listed in Table 2. To quantify the variations of resistance prevalence as a function of the parameter values (sensitivity analysis) we ran the model with 3 different values for each parameter (varying it to 90%, 100% and 110% of their best estimate value), singularly and in pairs to estimate possible synergistic effects (amounting to 1806 simulations of the model). The predicted resistance prevalence was the result of an average over a period of 400 days.To quantify synergistic effects, we define the cooperation coefficient (CC) for each couple of parameters as:

$$CC=\sqrt{\frac{{\left(R\left({p}_{\mathrm{i}}\&{p}_{\mathrm{j}}\right)-R\left(pre\right)\right)}^{2}}{{\left(R\left({p}_{i}\right)-R\left(pre\right)\right)}^{2}+{\left(R\left({p}_{\mathrm{j}}\right)-R\left(pre\right)\right)}^{2}}}$$

where “R (pre)” is the baseline prevalence, “R(p_i_)”, “R(p_j_)” and “R(p_i_&p_j_)” represent the resistance prevalence when changing the first , the second and both parameters, respectively. According to this definition, a CC > 1 implies that the coupled parameters have a synergistic effect as their combined effect is greater than the sum of the single effects. Before calculating CC, we combined the pairs of interventions in such a way that they both resulted in either a decrease (boosted interventions) or an increase (reduced interventions) in hospital resistance.

## Results

### Literature review

Out of 86 studies, 31 systematic reviews (SRs) were considered eligible for final inclusion (Supplementary Fig. 2). Twenty-three SRs (74%) were published in the last 5 years. Effectiveness of IPC and AMS interventions were considered separately in 18 (58%,) and 10 (32%) SRs respectively, while only 3 (9%) SRs considered the impact of both AMS and IPC on MDR bacteria ecology. When considering specific AMS and/or IPC interventions, 14 SRs (45%) reported the measured impact of specific single policy (e.g. antibiotic cycling and decolonisation) on targeted pathogen (e.g. MRSA). Fourteen SRs (45%) provided a descriptive impact of interventions. Eighteen SRs (58%) assessed MDR variation by AMS and/or IPC, 14 of which were able to give specific information per single pathogen of interest. Nineteen (45%) SRs focused on at least one Gram-positive bacteria (18 on MRSA and 8 on VRE); 8 focused on at least one Gram-negative bacteria (6 on CRPA, 6 on CRAB, 2 on CRKP, and 3 on CRE). MRSA, VRE, CRPA, and CRAB were the most frequently analysed bacteria. Thirteen SRs (41%) considered other outcomes such as mortality (9/13; 69%), length of stay (8/13; 61%), cost saving (7/13; 53%), reduction in antibiotic prescription (5/13; 38%) and nephrotoxicity (1/13; 7%), *C. difficile* infection (7/13; 53%). Among them only 3 provided considerations on the impact of AMS and/or IPC on all the analysed outcomes. Five systematic reviews (16%) provided the effects of AMS and IPC interventions on CRKP or CRE incidence. Of those, two^4,26^ provided quantifiable information on AMS and IPC impact on colonisation and/or infection of CRKP, and three^6,27,28^ returned general information on MDR bacteria without specifically addressing CRKP. Three out of five considered AMS and^4,26,27^ and IPC effectiveness^6,26,27^; only one returned quantifiable data on both AMS and IPC.

Supplementary Tables 6 and 7 summarize the characteristics of the SRs and the measured impact of IPC and AMS interventions on CRKP and CRE epidemiology.

### Model

The pre-intervention period was characterized by a bed occupancy of 79%, an average prevalence of 7.0% and 5.7% of resistant and susceptible strains, respectively (Supplementary Table 5). During the post-intervention period, the bed occupancy was slightly less (71%), with an average CRKP prevalence of 5.8% and an average CSKP prevalence of 5.4% (Supplementary Table 5). Between pre- and post-intervention periods, the prevalence of susceptible strains did not differ significatively (Mann Whitney U test p = 0.17), while the resistance prevalence displayed a p value of 0.054. Days of therapy (DOTs) per 1000 pd decreased from 231 in the pre-intervention period to 146 in the post-intervention. The dosage of the different antibiotic classes changed as in Supplementary Table S4b, with an average relative risk reduction of acquiring resistance varying from 3.15 to 2.94, according to the risks reported in literature^29^. Following the methods explained in Supplementary Table S3, the decrease in both DOTs and relative risk leads to an overall decrease in the transmission probability by antibiotic consumption from A^PRE^ = 1.50 to A^POST^ = 1.28.

**Figure 2:**
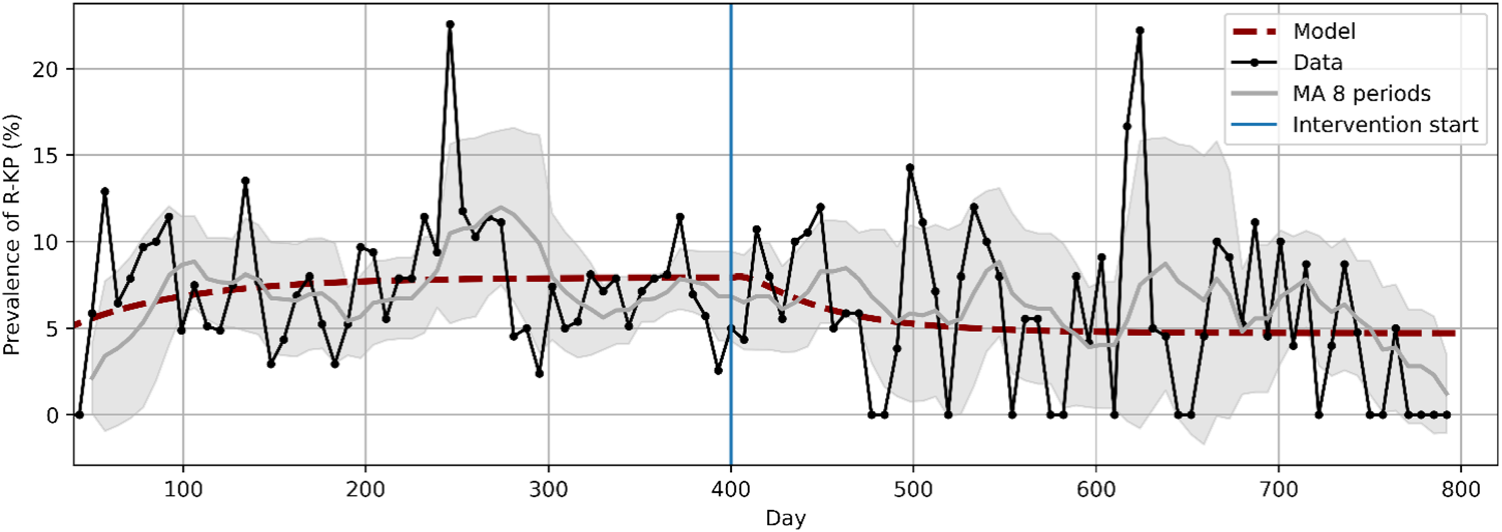
CRKP weekly point prevalence data and model estimates over time. Data is plotted both as raw data and as a moving average over 8 time points with the standard deviation as confidence interval (grey area). Model predictions are shown as a dashed red line.

Figure 2 shows the estimated percentage of CSKP and CRKP-positive patients over time as weekly point prevalence, both for real and model data.

The model predicts an average resistance prevalence of 7.1% and 5.2%, in fair agreement with the observed values (7.0% and 5.8%). The estimated values of hand hygiene compliance (h) and cohorting (q), which have been fitted both in the pre- and post-intervention periods, did not change significantly (q_PRE_ = q_POST_ = 0,10 ± 0,07, h_PRE_ = 0,855 ± 0,008 and h_POST_ = 0,868 ± 0,008), even if no constraints were applied to obtain these results. We could therefore assume that reduction of antibiotic consumption (A), the only variable that changed from pre- to post-intervention, was sufficient to explain the decrease in resistance prevalence.

### Sensitivity analysis

**Figure 3:**
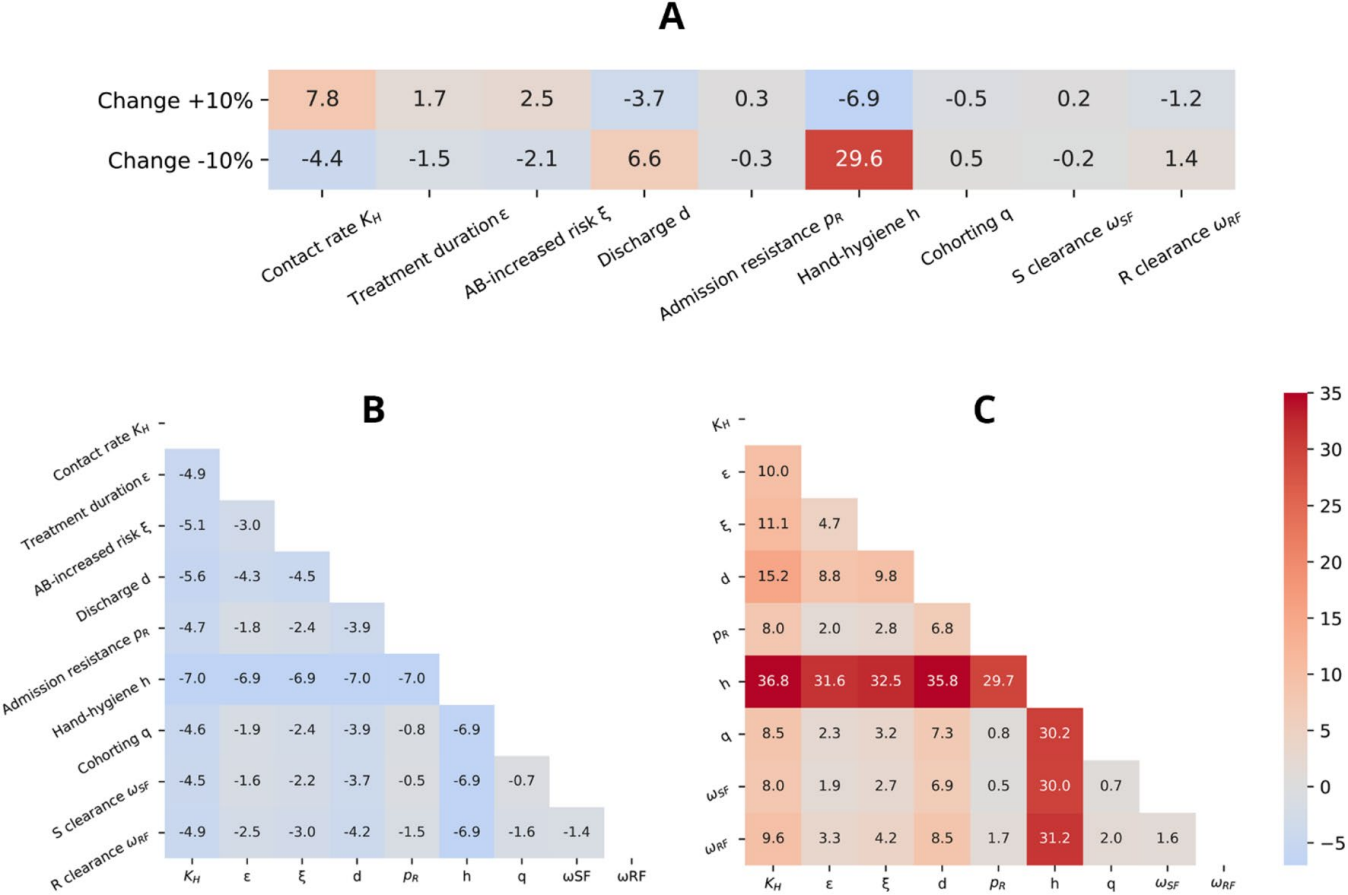
Simulation of the effect of (**A**) single interventions, (**B**) pairs of boosted interventions and (**C**) pairs of reduced interventions. The effects are estimated as difference in resistance prevalence (%) compared to the prevalence observed before intervention, calculated assuming an increase or decrease of 10% for each parameter. The color scale (centered at 0%) goes from decreased prevalence (blue) to increased prevalence (red). *K*_*H*_: per-capita contact rate (daily contacts per HCW per patient); ε: treatment duration (LOS fraction); ξ: increased risk of acquiring resistance for antibiotic (AB) pressure; *d*: average discharge rate (inverse of LOS); *p*_*R*_: resistance prevalence at admission; *h*: hand hygiene compliance (probability of correct hand washing after contact); *q*: fraction of HCWs allocated to cohorting (removed from the population); ω_*SF*_: clearance rate of colonized by susceptible strains; ω_*RF*_: clearance rate of colonized by resistant strains.

**Figure 4:**
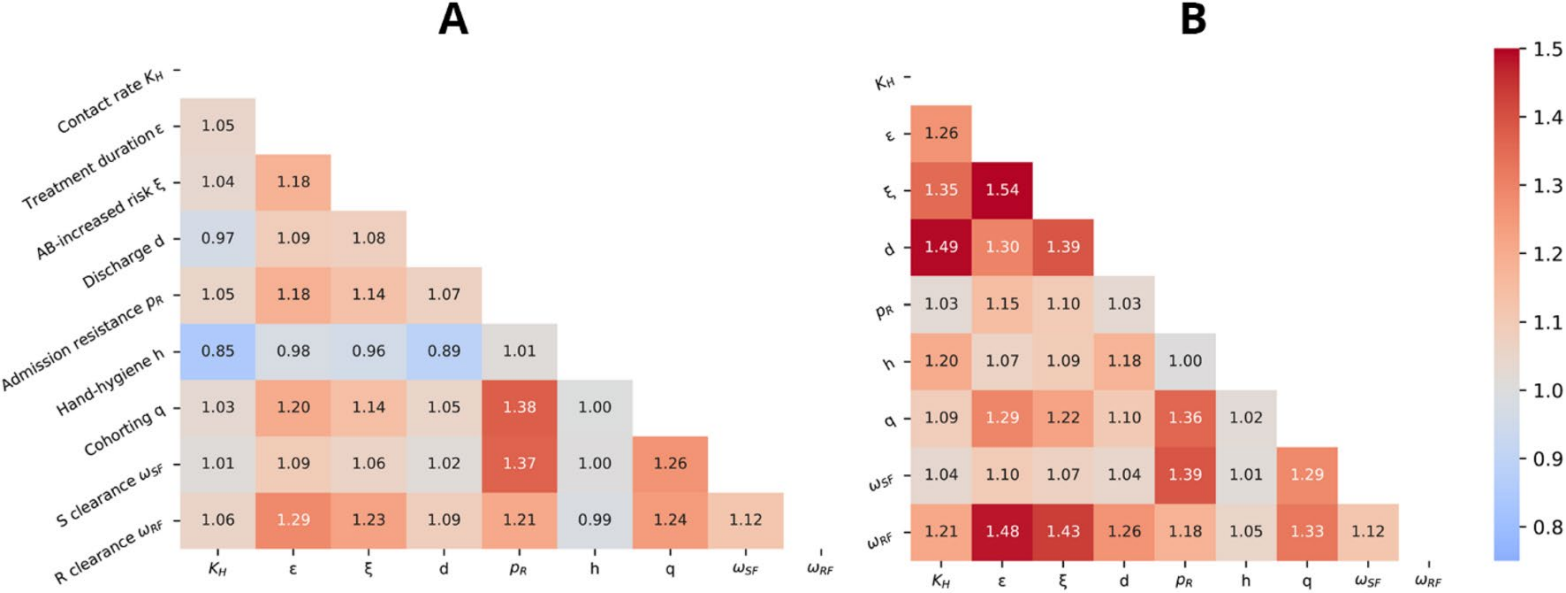
Cooperation coefficient of paired parameters in case of their implementation (**A**) and reduction (**B**). The color scale (centered at CC = 1) goes from uncooperative effect (blue) to cooperative effect (red). *K*_*H*_: per-capita contact rate (daily contacts per HCW per patient); ε: treatment duration (LOS fraction); ξ: increased risk of acquiring resistance for antibiotic (AB) pressure; *d*: average discharge rate (inverse of LOS); *p*_*R*_: resistance prevalence at admission; *h*: hand hygiene compliance (probability of correct hand washing after contact); *q*: fraction of HCWs allocated to cohorting (removed from the population) ; ω_*SF*_: clearance rate of colonized by susceptible strains; ω_*RF*_: clearance rate of colonized by resistant strains.

The effect of single interventions on CRKP prevalence over time were further analysed (Fig. 3, 4 and Supplementary Fig. 3). Figure 3 shows the effects of a ± 10% change in single (Fig. 3a) and paired parameters (Fig. 3b, c) with respect to the pre-intervention values.

The most effective parameter is (h), related to hand hygiene compliance, which led to a 7% reduction in resistance prevalence if increased by 10%. In general, the results of parameters variation are not symmetric as shown in Fig. 3a.

Regarding pairwise variation of parameters (Fig. 3b, c), when coupled with hand hygiene, none of the parameters could further decrease the resistance prevalence. Except hand hygiene intervention, the largest effects (about 5% reduction in resistance prevalence) were observed recombining the contact rate (K_H_)—a parameter influenced by isolation—with the discharge rate (d) or with the (ξ) parameter (risk of acquiring resistance due to antibiotic pressure).

We also investigated which pair of interventions have the most synergistic effect (Fig. 4).

A value of CC = 1.38 was observed when both cohorting (q) and screening at admission (p_R_) were increased, and CC = 1.37 when screening at admission (p_R_) was increased together with a higher clearance rate of CSKP colonization (${\omega }_{SF}$). However, in both cases, the overall reduction in resistance prevalence is about ten times lower than the one obtained for hand hygiene intervention alone (−0.8% and −0.5% in Fig. 3b versus −6.9% in Fig. 3a). By contrast, the maximum synergistic effect causing an increase in resistance prevalence was observed when (i) the treatment duration (ε) was increased together with an increase of the resistance risk (ξ) (CC = 1.54) or with a decrease of the clearance rate of resistant colonization (${\omega }_{RF}$) (CC = 1.48), or (ii) when the length of stay (d) together with a lower level of patient isolation (K_H_) were combined (CC = 1.49). Once again, we observed that the combined effects were lower than just reducing hand hygiene compliance alone (respectively, an increase of 4.7%, 3.3% or 15.2% in prevalence in Fig. 3c compared to the 29.6% increase due to reduction of hand hygiene compliance in Fig. 3a).

## Discussion

Major AMS goals are the optimization of patients’ care, which implicitly includes appropriateness of antimicrobial therapy and avoidance of unintended consequences, containment of healthcare costs, as well as education of frontline prescribers. In the context of AMS, information technology has been used to make guidance documents more accessible, to assess quality indicators, to measure antibiotic consumption, to predict infections and resistance^30–32^. Many guidelines are available, even though not all the recommendations are always applicable due to limited infrastructures, personnel, budget and adequate technical and diagnostic resources^33–35^. The aim of this study was to create a model to assess the impact of different AMS and IPC interventions in different epidemiological scenarios, in order to provide a practical advice on which interventions could be prioritized in an AMS programme, considering both the hospital setting and the local resources.

This study addressed AMS through a mathematical model developed using literature data and calibrated with real data on effectiveness of an advanced AMS and IPC programme implemented in a setting with a high rate of antimicrobial resistant infections^24^. Three crucial issues, that could lead to a lower reliability of mathematical models in real life scenarios, were considered. Firstly, models may miss to consider drug-susceptible and drug-resistant strains separately^18^, thus we considered three different populations: uncolonized, and colonized/infected by both susceptible and resistant strains. Since the frequency of susceptible-resistant transitions and within host susceptible-resistance coexistence are usually unknown or not measurable^18^, we did not explicitly model these events. Thus, the simultaneous presence of patients colonized by resistant or susceptible strains is mainly due to the influx of both types from the community outside the hospital. Secondly, multiple models simulate the effect of an increased resistance risk^10^ or bacterial transmissivity ^[^^16^^]^, but do not provide a mechanistic interpretation of the parameters change due to the implementation of AMS or IPC interventions. We overcame this limit by providing a rational link between all the parameters of the model and literature/clinical data (gel consumption, DOTs, increased risk). Finally, models may lack external validation on longitudinal epidemiological data, thus we used available data on resistance prevalence from a longitudinal study to validate the model in a real intervention scenario.

The model was fed with clinical and literature data, fitting the missing parameters on the longitudinal time series of point prevalence of our target ward. We then validated the model describing the changes in resistance prevalence in a real case study based on the decrease of antibiotic consumption. We showed that the restriction in antibiotic consumption, imposed to the model accordingly to the observed DOTs, was able to reproduce the point prevalence data. Even if some unobserved parameters (hand hygiene compliance and cohorting) have been fitted to the data, they did not change significantly from pre- to post-intervention, suggesting that the change in the model outputs were due only to the intervention on antibiotic prescription and no significant overfitting data occurred.

To further understand the impact on transmission of AMS interventions, we run a sensitivity analysis to evaluate which interventions (implemented singularly or in combination) could mostly affect the resistance prevalence in our setting. Hand hygiene compliance resulted the most relevant intervention influencing the resistance prevalence, followed by the contact rate and discharge rate. None of the combinations considered in the sensitivity analysis overcame the effectiveness of hand hygiene compliance alone in reducing the resistance prevalence. Nonetheless, an effect of around 5% was achieved by pairing the contact rate with the discharge rate or with the increased risk of acquiring resistance due to antibiotic pressure. This prediction can be of practical use when other interventions cannot be implemented in a specific setting. Other synergistic effects were observed: for example, prolonged antibiotic treatment together with higher resistance risk produced an increase in resistance prevalence 4.7%. We remark that an increase of the discharge rate of colonized patients might potentially increase pathogen transmission in the community, however the risk of an increased prevalence in that setting could be partially mitigated by the loss of carrier status over time.

Taken all together, these results can have relevant implications on clinical practice. In the perspective of starting or implementing an AMS programme, by providing the required data of the target ward, the model can produce tailored estimates on the most effective strategies for a specific setting.

We acknowledge that our work may have some limitations. Environmental contribution and patient-to-patient transmission were not modelled. Data used for validation are reasonably stationary and do not show significant trends (thus they represent an endemic infection condition), so that we could not validate the ability of the model to describe an emerging outbreak. Given the low numbers of infected patients, the choice of a stochastic approach could have been more informative in terms of model variability^36^, but for the sensitivity analysis we performed, considering all the model parameter variations singularly and in pairs, the computational burden to evaluate the confidence interval for each scenario would have been unfeasible. We suggest for future studies to apply downstream stochastic simulations once a limited list of scenarios of interest has been identified for the specific use case. Furthermore, our clinical data combined samples of both colonization and infection cases thus possibly over-estimating the percentage of resistant pathogens at admission, as it’s more likely that a microbiological sample could have been collected from a patient with a suspected infection. Additionally, in our model the effect of antibiotic pressure on selecting resistance is implemented as a multiplicative factor in the transmission probability, as in Austin et al.^16^. A possible extension would be to consider mechanisms for CRKP to develop endogenously (e.g. co-resistance), for example by including a transition from the P_S_ to the P_R_ compartment. Finally, our data included 8 weeks of the initial SARS-CoV-2 pandemic period, which may have added confounding facots, but we did not observe significant changes in screening strategies or clinical specimen collections.

Future directions of this work should focus on testing the model in different hospital settings, including high-risk wards (e.g. transplants or haematologic units) and further validate the effect of other interventions, singularly or in combination. Such predictions could be used as supporting material for the implementation of stewardship programmes. Moreover, if data were available, the model could be further expanded to represent more detailed clinical aspects, such as the stratification of patients into infected and colonized groups. In the present study, the analysis and parametrization has been presented for CRKP, but the modelling framework can be applied to other MDR pathogens by an appropriate choice of the parameter values. In that case, parameters like probability of bacterial transmission from patients to HCWs (α) and vice-versa (γ), the increased risk of acquiring resistance for antibiotic pressure (ξ) and eventually the time frequency of active decolonization (ω) (the latter not considered for CRKP) should be modified accordingly. If available, pathogen genotyping data could provide information on evolution/transfer of specific genetic determinants. A further step to add complexity to the model can be the simulation of screening procedures and patient flow between hospital wards, together with predictions of hospital costs required by implementing a particular intervention. The aforementioned framework would contribute to pursuing a model increasingly reflecting real life scenarios, and with a predicting ability encountering the practical demands of stewardship or infection control programmes.

### Supplementary Information

Supplementary Information.

## Data Availability

The clinical data utilized for model validation have been shared in an aggregated fashion within the main document and the Supplementary Information file.

## Code availability

The code related to the model implementation and sensitivity analysis is shared at https://github.com/FraDurazzi/MARTHE.
