# Author Correction: Modelling antimicrobial resistance transmission to guide personalized antimicrobial stewardship interventions and infection control policies in healthcare setting: a pilot study

**DOI:** 10.1038/s41598-023-51051-x

**Published:** 2024-01-09

**Authors:** Francesco Durazzi, Maria Diletta Pezzani, Fabiana Arieti, Omar Simonetti, Lorenzo Maria Canziani, Elena Carrara, Lorenzo Barbato, Francesco Onorati, Daniel Remondini, Evelina Tacconelli

**Affiliations:** 1 Division of Infectious Diseases, Department of Diagnostics and Public Health, University of Verona, Verona, Italy; 2 Department of Physics and Astronomy, University of Bologna, Bologna, Italy; 3 Infectious Diseases Unit, University Hospital, Trieste, Italy; 4 Department of Pharmacy, Azienda Ospedaliera Universitaria Integrata Verona, Verona, Italy; 5 Department of Cardiac Surgery, Verona University Hospital, Verona, Italy

Correction to: *Scientific Reports* 10.1038/s41598-023-42511-5, published online 22 September 2023

The original version of this Article contained an error in the Acknowledgements section.

“This project has been partially supported by: (1) EPI-Net project funded by the COMBACTE-MAGNET and ECRAID-Base consortia. EPI-Net receives financial support from Innovative Medicines Initiative 2 Joint Undertaking under grant agreement No. 115737, resources of which are composed of financial contribution from the European Union Seventh Framework Programme (FP7/2007-2013) and European Federation of Pharmaceutical Industries and Associations (EFPIA) companies’ in-kind contribution, and the European Union Horizon 2020 research and innovation programme under grant agreement No. 965313, respectively. (2) European Union Horizon 2020 research and innovation programme under grant agreement No. 874735 “Versatile emerging infectious disease observatory—forecasting, nowcasting and tracking in a changing world (VEO)”.”

now reads:

“This project has been partially supported by: (1) ECRAID-Base project has received funding from the European Union’s Horizon 2020 research and innovation programme under grant agreement No. 965313. COMBACTE-MAGNET project received support from the Innovative Medicines Initiative Joint Undertaking under grant agreement No. 115737 which is composed of financial contribution from the European Union Seventh Framework Programme (FP7/2007-2013) and EFPIA companies in kind contribution. (2) European Union Horizon 2020 research and innovation programme under grant agreement No. 874735 “Versatile emerging infectious disease observatory—forecasting, nowcasting and tracking in a changing world (VEO)”.”

The original Article has been corrected.

