# Supplementary material for: Modelling antimicrobial resistance transmission to guide personalized antimicrobial stewardship interventions and infection control policies in healthcare setting: a pilot study
Source: Sci Rep. 2023 Sep 22;13:15803. doi: 10.1038/s41598-023-42511-5 (PMC10516989; doi:10.1038/s41598-023-42511-5)
Supplement: Supplementary file 1 — Supplementary Information. [file 41598_2023_42511_MOESM1_ESM.pdf]

Supplementary Information - Modelling antimicrobial resistance transmission to guide personalized antimicrobial stewardship interventions and infection control policies in healthcare setting: a pilot study.

Scoping review

Supplementary Table S1. Search terms used for the scoping review

Pathogen search terms:	"Enterobacteriaceae Infections"[Mesh] OR "Klebsiella Infections"[Mesh] OR "Klebsiella"[Mesh] OR "Klebsiella pneumoniae"[Mesh] OR "Carbapenem-Resistant Enterobacteriaceae"[Mesh] OR "Pseudomonas aeruginosa"[Mesh] OR "Pseudomonas Infections"[Mesh] OR "Acinetobacter baumannii"[Mesh] OR "Acinetobacter Infections"[Mesh] OR "Staphylococcal Infections"[Mesh] OR "Staphylococcus"[Mesh] OR "Staphylococcus aureus"[Mesh] OR "Methicillin-Resistant Staphylococcus aureus"[Mesh] OR "Enterococcus"[Mesh] OR "Vancomycin Resistance"[Mesh])
Intervention search terms:	"Cross Infection/prevention and control"[Mesh] OR "Health Facilities"[Mesh]) AND ("Bacterial Infections/prevention and control"[Mesh] OR "Bacterial Infections/drug therapy"[Mesh] OR "Anti-Bacterial Agents"[Mesh] OR "Drug Prescriptions/statistics and numerical data"[Mesh] OR "Behavior Therapy/methods"[Mesh] OR "Disinfectants/administration and dosage"[Mesh]) AND ("Cross Infection/transmission"[Mesh] OR "Drug Resistance, Microbial"[Mesh] OR "Drug Resistance, Multiple"[Mesh]

Supplementary Table S2. List of the retrieved variables from the systematic reviews

Variable	Description
Article	Title, first author
Year	Year
Article type	To clarify the type of article, e.g. systematic review, meta-analysis
Number of articles included	To clarify how many articles are included in the systematic review
Year data	Year/years to which data refer to
Setting	Type of setting from which data were
Interventions	Type of antimicrobial stewardship or infection control interventions
Pathogen	Resistant pathogens for which effectiveness of interventions were analysed
Parameters	Parameters utilized in the article to assess the intervention effectiveness, e.g. Incidence ratio (IR), odds ratio (OR), risk ratio (RR), risk difference (RD)
Impact of interventions	To clarify whether the impact of a specific intervention on a specific pathogen was calculated

Model

Supplementary Table S3. List of interventions and description of related model parameters

Cohorting	Cohorted contacts reduces HCW-patient mixing, by reducing the number of HCWs contributing to transmission [1]. For example, one-to-one nursing by a fraction of HCWs ($H = H_F+H_S+H_R$) corresponds to an effective reduction H(1-q) in HCWs number in the model.
Isolation and pre-emptive isolation	HCW-patient mixing can be decreased by reducing the number of daily contacts between HCWs and patients, through the respective parameter K_H.
Antibiotic consumption policies	By reducing the antibiotic DOTs or choosing antibiotics with lower risk of selecting resistant strains [1,2], it is possible to decrease the emergence and spread of resistant strains. In their study, Austin *et al* antibiotic restriction policies are introduced into the model to simulate reduction in selection pressure (and hence probability of patient colonization) [1]. They estimate that, if antibiotic selection pressure gives an increased relative risk ξ of acquisition whist the patient is receiving treatment, and patients receive antibiotics for a fraction ϵ of their LOS, then the probability per contact of colonization is increased by a factor of $A = 1 + \epsilon(\xi - 1)$. Within our case study, we estimate ϵ from the days of therapy (DOT) per pd of the resistance selecting antibiotics, as: $$\epsilon = \frac{avg.treatment\ duration}{LOS} = \frac{DOT\ per\ pd\ *\ pd\ /\ admitted\ patients}{LOS} = \frac{DOT\ per\ pd\ *\ LOS}{LOS} = DOT\ per\ pd = 0.231$$ where the average treatment duration is meant to be as if the daily doses observed had been distributed to all the patients admitted, thus it must not be confused with the average treatment duration calculated only on the patients who received an antibiotic treatment. The increased relative risk estimate is $\xi = 3.15$ for the pre-intervention period and $\xi = 2.94$ for post-intervention, as the average of the resistance selecting antibiotics increased risks from [6]. The average is computed on literature risks weighted on hospital data DOTs of β-lactam, cephalosporins, carbapenems and fluoroquinolones antibiotics. Antibiotic prescription for the

	patients was considered as independent from the epidemiological status, in the sense that the DOTs were considered to be the same for each epidemiological compartment (P_F, P_S, P_R).
Hand hygiene	Hand hygiene compliance h contributes to the probability of bacterial transmission during the contacts between contaminated and un-colonized individuals [3]. To estimate h, we consider the following equation:<br>$$h = \frac{C_h}{C} = \frac{\#\ of\ contacts\ followed\ by\ sanitizations}{\#\ of\ contacts}$$<br>In particular, we can estimate:<br>$$C_h = \#\ of\ contacts\ followed\ by\ sanitization = total\ gel\ consumption\ /\ single\ gel\ dose$$<br>In which the recommended single gel dose is 0.004 litres, as indicated in the WHO Guidelines on Hand Hygiene in Health Care.<br>Since we don't know the total amount of gel consumption, we can estimate it from our data as follows:<br>$$total\ gel\ consumption = gel\ consumption\ per\ pd\ *\ pd$$<br>where the gel consumption per pd is 0.04427 litres per patient-days (*pd*). Patient days (*pd*, with values in Supplementary Table S5) are defined as the sum of the LOS of all patients admitted in the observation period (equal to 14382 *pd* in the pre-intervention period).<br>The total number of contacts C can be estimated as:<br>$$C = \#\ of\ contacts = K_H * H * P * days$$<br>Where K_H is the number of daily contacts per HCW per patient (Table 1), H=17 is the HCWs number, P = 0.79 bed occupancy * 46 beds = 36.34 is the average number of patients, and *days*=399 is the duration of the pre-intervention period.<br>Thus, we can calculate h as follows:<br>$$h = \frac{C_h}{C} = \frac{gel\ consumption\ per\ pd\ *pd\ /\ single\ gel\ dose}{K_H * H * P * days}$$<br>In our case study, both h and K_H must be estimated, but through this relation, only one need to be fitted.
Screening at admission	Universal screening was modelled through the parameter describing the resistance prevalence at admission as it usually results in patient isolation thus decreasing the entry of individuals colonized/infected with resistant strains. The fraction of patients colonized and or infected at admission had been extracted from the hospital data. To simulate the effect of the screening at admission, followed by isolation, we decreased or increased this rate of infected people at admission.

Clinical data (SAVE intervention)

To estimate prevalence of *Klebsiella pneumoniae* samples collected within the 72 hours from admission and on weekly basis were selected. The samples comprised rectal swabs from screening activities and clinical specimens from different sources collected at the discretion of the attending physicians (e.g. blood, wound swabs, urine, sputum, bronchoalveolar lavage). Patients colonized and/or infected by carbapenem resistant *K.pneumoniae* (CRKP) were those with a sample positive for carbapenem resistant strain; patients colonized and/or infected by carbapenem susceptible strain (CSKP) were those with a sample holding a negative result for CRKP (*e.g.* samples of *Klebsiella pneumoniae* ESBL-producers were considered in this category); uncolonized or "free" patients were defined as those with negative microbiological samples or positive for pathogens other than *K. pneumoniae*. AMC data (including defined daily dose-DDD and days of therapy-DOT) were collected for a list of antibiotics for which exposure has been associated with the development of the carbapenem-resistance: carbapenems (ertapenem, meropenem, imipenem/cilastatin), betalactams-betalactamases inhibitors combinations (BLBLI) (amoxicillin-clavulanate and piperacillin-tazobactam), third and fourth generation cephalosporins (ceftazidime, ceftriaxone, and cefepime), and fluoroquinolones (ciprofloxacin and levofloxacin) [2]. Bed occupancy, number of admissions, length of hospital stay, staffing (nurses) levels were also recorded (Tables 5, 6, 7).

Supplementary Table S4. A) Variables collected for model validation. AMC: antimicrobial consumption; DDD: defined daily dose; DOT: days of therapy; HCW: health care worker; PF: not colonized/free; PR: colonized/infected by resistant strain; PS: colonized/infected by susceptible strain. B) DOTs per patient-day for the different antibiotic classes.

(a)

**Variable**	**Description**
Prevalence on admission	Percentage of colonized/infected patients (S and R) at admission
Weekly point prevalence	Percentage of colonized/infected (S and R) patients hospitalized at time of data collection
Number of beds	Number of beds available in the ward considered
Length of stay for P_F- P_S- P_R	Average days spent in the hospital by patients
HCW to patient ratio	Number of HCW in relation to the number of patients.
Patient days	Total number of days spent by patients in the hospital
Number of admissions	Total number of patients hospitalized in the time period considered

AMC data	DDDs and DOTs of antibiotics associated to carbapenem-resistance development

(b)

Antibiotic class	DOTs per 1000 pd pre-intervention	DOTs per 1000 pd post-intervention
Penicillins	100.46	90.23
Cephalosporins	47.66	28.87
Carbapenems	61.05	19.93
Fluoroquinolones	21.64	6.48

Supplementary Table S5. Summary of SAVE data from pre and post-intevention periods. CSKP:arbapenem-susceptible Klebsiella pneumoniae; CRKP:c arbapenem-resistant Klebsiella pneumoniae; DOT:; KP: Klebsiella penumoniae.

**Variables**	**Pre-intervention N (%)**	**Post-intervention N (%)**
**Prevalence on admission**		
Total isolates (<72h from admission)	883 (100%)	223 (100%)
CSKP	22 (2.5%)	3 (1.3%)
CRKP	5 (0.6%)	16 (7.2%)
Free KP	856 (96.9%)	204 (91.5%)
**Weekly point prevalence**	**Pre-intervention (N)**	**Post-intervention (N)**
CSKP	117	62
CRKP	139	69
Free KP	1760	1061
**CRKP prevalence**	7.0%	5.8%
Mean CRKP/week	2.4	1.2
**Length of stay**	**Pre-intervention days (d)**	**Post-intervention days (d)**
CSKP	32.4 d	34 d
CRKP	60 d	45 d
Free KP	10,05 d	8,69 d
**Ward data**	**Pre-intervention (N)**	**Post-intervention (N)**
Number of beds	46	46
Number of nurses/patient ratio	6,2	6,2
Bed occupancy	79%	71%
Admissions	1357	1421
Patient days	14382	13008
Total consumption of alcohol gel	357,6	446,9
Alcohol gel consumption per 1000 patient days	24,19	34,36
Antibiotic consumption DOT per 1000pd	231	146

Supplementary Figure S1. CRKP weekly point prevalence over time, plotted both as raw data and as a moving average on 8 periods-weeks with the standard deviation as confidence interval. Dashed lines represent the average resistance prevalence before (light blue) and after (dark blue) the intervention.

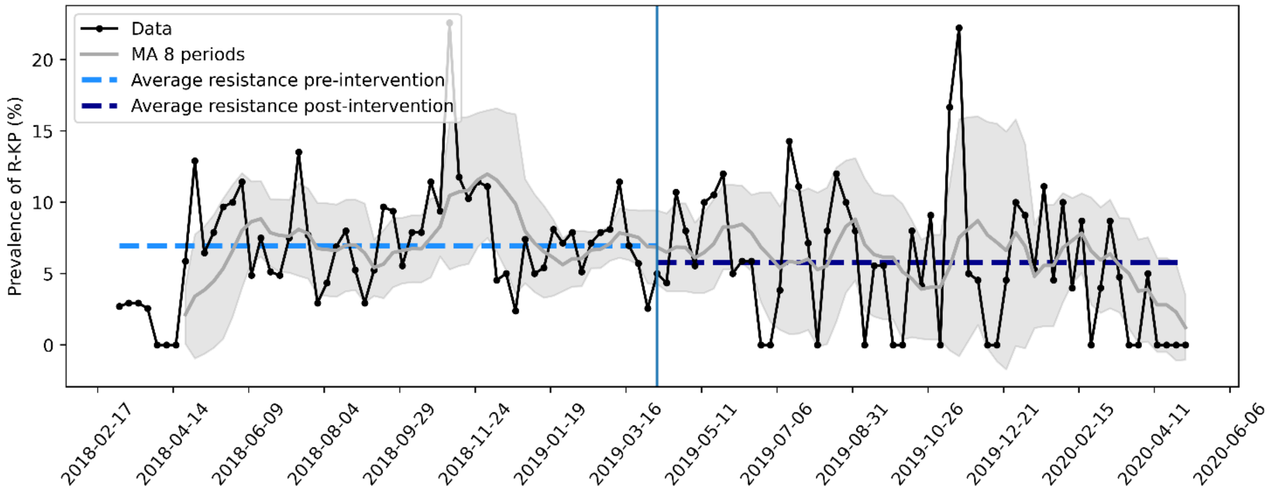

Supplementary Figure S2. Flow chart of the scoping review.

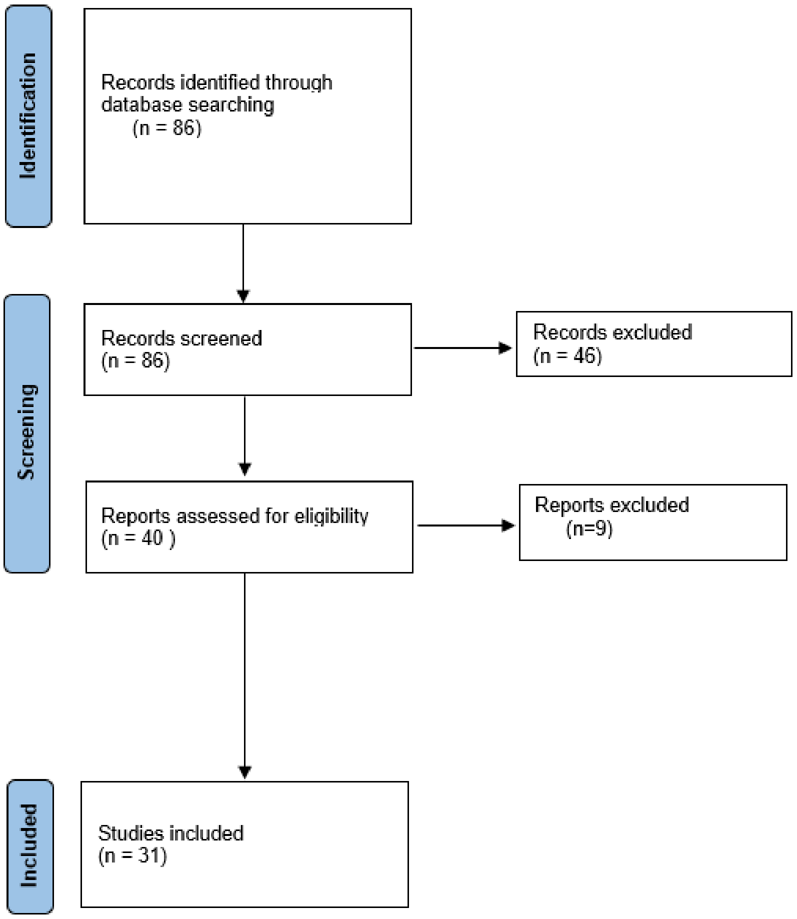

Supplementary Table S6. List of publications analysing the impact of Infection Prevention and control (IPC) or Antibiotic stewardship (AMS) interventions included in this study. SR: systematic review; MA: meta-analysis; LTCF: Long-term care facilities; MRSA: Methicillin-resistant Staphylococcus aureus; MDRO: Multidrug-resistant organisms; Vancomycin-resistant Enterococci (VRE); CDI: Clostridium difficile infection; CRE: Carbapenem-resistant Enterobacterales; CRAB: Carbapenem-resistant Acinetobacter baumannii; CRPA: Carbapenem-resistant Pseudomonas aeruginosa; DDD: defined daily dose; ESBL: Extended spectrum beta-lactamase; NA: Not available; Trend: Range of change in slope of the outcome between pre- and post-intervention; IC: Immediate change in the level of outcome between pre- and post-intervention; LOS: Length of stay; RR: Pooled risk ratio, reduction in colonization and/or infection rate after intervention; RaRa: Pooled rate ratio, rate ratio of infections and/or colonisation between standard of care and intervention period; IRaRa: Incidence rate ratio, incidence rate ratio of infections and/or colonisation between standard of care and intervention; IRs: Incidence ratio, ratio between infection/colonisation before and after intervention; %r: Percentage of reduction, reduction expressed in percentage of specific infection caused by a specific pathogen; IRD: Incidence rate difference, difference in incidence rate per 1000 patient days of resistant bacteria; OR: odds ratio, change in incidence of infection and/or colonisation.

First author Year of publication	Study type	Setting	Intervention	Pathogen	Parameters	Other outcomes
Tomczyk S. 2019 [5]	SR	Healthcare	IPC	CRE, CRAB, CRPA	Trend, IC	
Lee M.H. 2019 [5]	SR	LTCF	IPC	MDRO	Descriptive	
Fan C.Y. 2019 [6]	MA	Hospital	IPC	CRAB	RR	
Chang N.C.N. 2019 [8]	SR, MA	Healthcare	IPC	MRSA, VRE	IRaRa	CDI
Nathwani D. 2019 [9]	SR	Hospital	AMS	MDRO	Descriptive	LOS, mortality, costs
Bertollo L.G. 2018 [10]	SR	Hospital	AMS	MDRO	Descriptive	LOS, mortality, DDD, costs, CDI
Moralejo D. 2018 [11]	SR	Healthcare	IPC	MRSA	Descriptive	
Baur D. 2017 [12]	SR, MA	Hospital	AMS	MRSA, VRE, ESBL	IRs	CDI
Davey P. 2017 [13]	SR, MA	Hospital	AMS	MDRO	RD	LOS, CDI
Teerawattanapong 2017 [14]	SR, MA	Hospital	IPC and AMS	ESBL, CRE, CRAB, CRPA	RaRa	Mortality
Honda H. 2017 [15]	SR, MA	Healthcare	AMS	MRSA, ESBL, CRAB, CRPA	ARD	LOS, mortality, DDD, costs
Marra A.R. 2017 [16]	SR, MA	Hospital	IPC	MRSA, VRE	RR	CDI
Van Dijck C. 2017 [17]	SR	Hospital	AMS	MDRO	Descriptive	Mortality, DDD
Kizny Gordon A.E. 2017 [18]	SR	Healthcare	IPC	CRE, CRAB, CRPA	Descriptive	

Gould D.J. 2017 [19]	SR, MA	Healthcare	IPC	MRSA	Descriptive	
Karanika S. 2016 [20]	SR, MA	Hospital	AMS	MRSA, ESBL, CRPA	%c, RD	LOS, Mortality, DDD, CDI
Nair R. 2016 [21]	SR, MA	Healthcare	IPC	MRSA	RR	Mortality
Frost S.A. 2016 [22]	SR, MA	Hospital	IPC	MRSA, VRE	IRR	CDI
Schuts E.C. 2016 [23]	SR, MA	Healthcare	AMS	MDRO	Descriptive	LOS, mortality, costs, nephrotoxicity
Campos A.C. 2016 [24]	SR	Healthcare	IPC and AMS	CRKP	Descriptive	
Kim H.Y. 2015 [24]	MA	Hospital	IPC	MRSA, VRE	RR	
Zaky A. 2015 [26]	SR	Hospital	IPC and AMS	MDRO	OR	LOS, mortality
López-Alcalde J. 2015 [27]	SR	Hospital	IPC	MRSA	na	
De Angelis G. 2014 [28]	SR, MA	Hospital	IPC	VRE	RR	LOS, mortality, costs
zur Wiesch P.A. 2014 [29]	SR, MA	Hospital	AMS	MDRO	IRD	
Kock R. 2014 [30]	SR	Hospital	IPC	MRSA	Descriptive	
Daneman N. 2013 [31]	SR, MA	Hospital	IPC	MRSA, VRE, ESBL	OR	
Hughes C. 2013 [32]	SR	LTCF	IPC	MRSA	na	
Chen A.F. 2013 [33]	SR	Hospital	IPC	MRSA	%r	Costs
Karki S. 2012 [34]	SR, MA	Healthcare	IPC	MRSA, VRE	IRaRa	
Kaki R. 2011 [35]	SR	Hospital	AMS	MRSA, ESBL	Descriptive	DDD, costs

Supplementary Table S7. List of publications analysing the impact of Infection Prevention and control (IPC) or Antibiotic stewardship (AMS) interventions limited to carbapenem resistant Klebsiella pneumoniae and carbapenem resistant Enterobacteriaceae. RR: Pooled risk ratio, reduction in colonization and/or infection rate after intervention; RaRa: Pooled rate ratio, rate ratio of infections and/or colonisation between standard of care and intervention period; IRaRa: Incidence rate ratio, incidence rate ratio of infections and/or colonisation between standard of care and intervention; IRs: Incidence ratio, ratio between infection/colonisation before and after intervention.

Intervention	Value	Indicator	First author, Year of publication
**Active AMS**	0.52	IR of infection or colonization per 1000 pd	Baur D, 2017 [11]
	0.39	RaRa of colonization, infection or acquisition	Teerawattanapong N, 2017 [13]
**Antibiotic cycling**	0,49	IR of infection or colonization per 1000 pd	Baur D, 2017 [11]
	-7,22	IRD per 1000 pd	Abel zur Wiesch P, 2014 [28]
**Antibiotic restriction**	0,77	IR of infection or colonization per 1000 pd	Baur D, 2017 [11]
**Audit and Feedback**	0,66	IR of infection or colonization per 1000 pd	Baur D, 2017 [11]
**IPC**	-0,01 to -4,81	Change in slope (ie, trend) between pre- and post-intervention	Tomczyk S, 2019 [4]
	-0,02 to -48,86	Change in level (ie, immediate change) between pre- and post-intervention	Tomczyk S, 2019 [4]
	0,17	RaRa of colonization, infection or acquisition	Teerawattanapong N, 2017 [13]
**Isolation**	-0,01 to -4,81	Change in slope (ie, trend) between pre- and post-intervention	Tomczyk S, 2019 [4]
	-1,19 to -48,86	Change in level (ie immediate change) between pre- and post-intervention	Tomczyk S, 2019 [4]
**Decolonisation**	0,44	RaRa of colonization, infection or acquisition	Teerawattanapong N, 2017 [13]
	0,45	RR for acquisition	Kim HY, 2016 [24]
**Hand hygiene**	-0,01 to -4,81	Change in slope (ie, trend) between pre- and post-intervention	Tomczyk S, 2019 [4]
	-0,02 to -48,86	Change in level (ie immediate change) between pre- and post-intervention	Tomczyk S, 2019 [4]
**Environmental cleaning**	0,38	RaRa of colonization, infection or acquisition	Teerawattanapong N, 2017 [13]
**Active surveillance**	-0,01 to -4,81	Change in slope (ie, trend) between pre- and post-intervention	Tomczyk S, 2019 [4]
	-0,02 to -48,86	Change in level (ie immediate change) between pre- and post-intervention	Tomczyk S, 2019 [4]
**Active AMS, IPC**	0.07	RaRa of colonisation, infection or acquisition	Teerawattanapong N, 2017 [13]

Supplementary Figure S3. Model predictions of CRKP prevalence over time (% of resistant patients w.r.t. total) when implementing interventions. Before day 400 is the pre-intervention period. After day 400, multiple scenarios are simulated corresponding to stricter interventions (3%, 5%, 10%, 15% stricter than the initial value). a) Cohorting level, b) hand hygiene, c) screening at admission aimed to reduce prevalence at admission, d) antibiotic restriction policies aimed to reduce treatment duration.

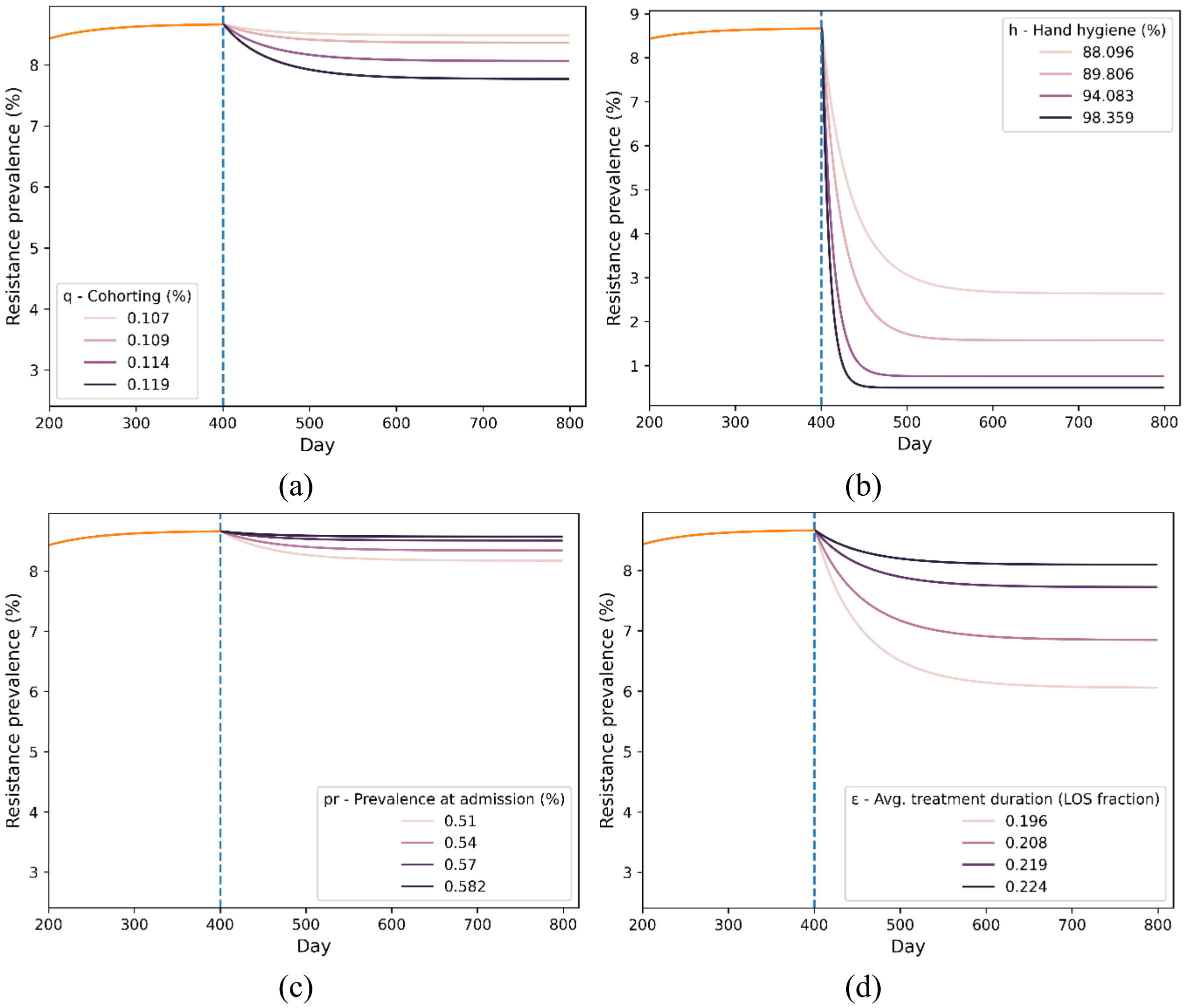

References

1. Austin, D. J., Bonten, M. J. M., Weinstein, R. A., Slaughter, S. & Anderson, R. M. Vancomycin-resistant enterococci in intensive-care hospital settings: Transmission dynamics, persistence, and the impact of infection control programs. *Proc. Natl. Acad. Sci.* **96**, 6908–6913 (1999).
2. Li, J., Li, Y., Song, N. & Chen, Y. Risk factors for carbapenem-resistant Klebsiella pneumoniae infection: A meta-analysis. *J. Glob. Antimicrob. Resist.* **21**, 306–313 (2020).
3. Sypsa, V. *et al.* Transmission Dynamics of Carbapenemase-Producing Klebsiella Pneumoniae and Anticipated Impact of Infection Control Strategies in a Surgical Unit. *PLoS ONE* **7**, e41068 (2012).
4. Tomczyk, S. *et al.* Control of Carbapenem-resistant Enterobacteriaceae, *Acinetobacter baumannii* , and *Pseudomonas aeruginosa* in Healthcare Facilities: A Systematic Review and Reanalysis of Quasi-experimental Studies. *Clin. Infect. Dis.* **68**, 873–884 (2019).
5. Lee, M. H., Lee, G. A., Lee, S. H. & Park, Y.-H. Effectiveness and core components of infection prevention and control programmes in long-term care facilities: a systematic review. *J. Hosp. Infect.* **102**, 377–393 (2019).
6. Fan, C.-Y. *et al.* Effect of chlorhexidine bathing on colonization or infection with Acinetobacter baumannii: a systematic review and meta-analysis. *J. Hosp. Infect.* **103**, 284–292 (2019).
7. Chang, N.-C. N. *et al.* Association between universal gloving and healthcare-associated infections: A systematic literature review and meta-analysis. *Infect. Control Hosp. Epidemiol.* **40**, 755–760 (2019).
8. Nathwani, D. *et al.* Value of hospital antimicrobial stewardship programs [ASPs]: a systematic review. *Antimicrob. Resist. Infect. Control* **8**, 35 (2019).
9. Bertollo, L. G., Lutkemeyer, D. S. & Levin, A. S. Are antimicrobial stewardship programs effective strategies for preventing antibiotic resistance? A systematic review. *Am. J. Infect. Control* **46**, 824–836 (2018).
10. Moralejo, D., El Dib, R., Prata, R. A., Barretti, P. & Corrêa, I. Improving adherence to Standard Precautions for the control of health care-associated infections. *Cochrane Database*

Syst. Rev. **2018**, (2018).

11. Baur, D. *et al.* Effect of antibiotic stewardship on the incidence of infection and colonisation with antibiotic-resistant bacteria and Clostridium difficile infection: a systematic review and meta-analysis. *Lancet Infect. Dis.* **17**, 990–1001 (2017).

12. Davey, P. *et al.* Interventions to improve antibiotic prescribing practices for hospital inpatients. *Cochrane Database Syst. Rev.* **2017**, (2017).

13. Teerawattanapong, N. *et al.* Prevention and Control of Multidrug-Resistant Gram-Negative Bacteria in Adult Intensive Care Units: A Systematic Review and Network Meta-analysis. *Clin. Infect. Dis.* **64**, S51–S60 (2017).

14. Honda, H., Ohmagari, N., Tokuda, Y., Mattar, C. & Warren, D. K. Antimicrobial Stewardship in Inpatient Settings in the Asia Pacific Region: A Systematic Review and Meta-analysis. *Clin. Infect. Dis.* **64**, S119–S126 (2017).

15. Marra, A. R., Schweizer, M. L. & Edmond, M. B. No-Touch Disinfection Methods to Decrease Multidrug-Resistant Organism Infections: A Systematic Review and Meta-analysis. *Infect. Control Hosp. Epidemiol.* **39**, 20–31 (2018).

16. Van Dijck, C., Vlieghe, E. & Cox, J. A. Antibiotic stewardship interventions in hospitals in low-and middle-income countries: a systematic review. *Bull. World Health Organ.* **96**, 266–280 (2018).

17. Kizny Gordon, A. E. *et al.* The Hospital Water Environment as a Reservoir for Carbapenem-Resistant Organisms Causing Hospital-Acquired Infections—A Systematic Review of the Literature. *Clin. Infect. Dis.* **64**, 1435–1444 (2017).

18. Gould, D. J., Moralejo, D., Drey, N., Chudleigh, J. H. & Taljaard, M. Interventions to improve hand hygiene compliance in patient care. *Cochrane Database Syst. Rev.* **2017**, (2017).

19. Karanika, S., Paudel, S., Grigoras, C., Kalbasi, A. & Mylonakis, E. Systematic Review and Meta-analysis of Clinical and Economic Outcomes from the Implementation of Hospital-Based Antimicrobial Stewardship Programs. *Antimicrob. Agents Chemother.* **60**, 4840–4852 (2016).

20. Nair, R. *et al.* Clinical Effectiveness of Mupirocin for Preventing *Staphylococcus aureus* Infections in Nonsurgical Settings: A Meta-analysis. *Clin. Infect. Dis.* **62**, 618–630 (2016).

21. Frost, S. A. *et al.* Chlorhexidine bathing and health care-associated infections among adult intensive care patients: a systematic review and meta-analysis. *Crit. Care* **20**, 379 (2016).

22. Schuts, E. C. *et al.* Current evidence on hospital antimicrobial stewardship objectives: a systematic review and meta-analysis. *Lancet Infect. Dis.* **16**, 847–856 (2016).

23. Campos, A. C. *et al.* Outbreak of Klebsiella pneumoniae carbapenemase–producing K pneumoniae: A systematic review. *Am. J. Infect. Control* **44**, 1374–1380 (2016).

24. Kim, H. Y. *et al.* The effects of chlorhexidine gluconate bathing on health care–associated infection in intensive care units: A meta-analysis. *J. Crit. Care* **32**, 126–137 (2016).

25. Zaky, A., Zeliadt, S. B. & Treggiari, M. M. Patient-Level Interventions to Prevent the Acquisition of Resistant Gram-Negative Bacteria in Critically Ill Patients: A Systematic Review. *Anaesth. Intensive Care* **43**, 23–33 (2015).

26. López-Alcalde, J. *et al.* Gloves, gowns and masks for reducing the transmission of meticillin-resistant Staphylococcus aureus (MRSA) in the hospital setting. *Cochrane Database Syst. Rev.* **2015**, (2015).

27. De Angelis, G. *et al.* Infection control and prevention measures to reduce the spread of vancomycin-resistant enterococci in hospitalized patients: a systematic review and meta-analysis. *J. Antimicrob. Chemother.* **69**, 1185–1192 (2014).

28. Abel zur Wiesch, P., Kouyos, R., Abel, S., Viechtbauer, W. & Bonhoeffer, S. Cycling Empirical Antibiotic Therapy in Hospitals: Meta-Analysis and Models. *PLoS Pathog.* **10**, e1004225 (2014).

29. Köck, R. *et al.* Systematic literature analysis and review of targeted preventive measures to limit healthcare-associated infections by meticillin-resistant Staphylococcus aureus. *Eurosurveillance* **19**, (2014).

30. Daneman, N., Sarwar, S., Fowler, R. A. & Cuthbertson, B. H. Effect of selective

decontamination on antimicrobial resistance in intensive care units: a systematic review and meta-analysis. *Lancet Infect. Dis.* **13**, 328–341 (2013).

31. Hughes, C., Tunney, M. & Bradley, M. C. Infection control strategies for preventing the transmission of meticillin-resistant *Staphylococcus aureus* (MRSA) in nursing homes for older people. *Cochrane Database Syst. Rev.* **2013**, (2013).

32. Chen, A. F., Wessel, C. B. & Rao, N. Staphylococcus aureus Screening and Decolonization in Orthopaedic Surgery and Reduction of Surgical Site Infections. *Clin. Orthop.* **471**, 2383–2399 (2013).

33. Karki, S. & Cheng, A. C. Impact of non-rinse skin cleansing with chlorhexidine gluconate on prevention of healthcare-associated infections and colonization with multi-resistant organisms: a systematic review. *J. Hosp. Infect.* **82**, 71–84 (2012).

34. Kaki, R. *et al.* Impact of antimicrobial stewardship in critical care: a systematic review. *J. Antimicrob. Chemother.* **66**, 1223–1230 (2011).
